# Disabled People’s Organisations increase access to services and improve well-being: evidence from a cluster randomized trial in North India

**DOI:** 10.1186/s12889-020-8192-0

**Published:** 2020-01-31

**Authors:** Nathan John Grills, Monsurul Hoq, Chun-Ping Pam Wong, Komal Allagh, Lawrence Singh, Fairlene Soji, G. V. S. Murthy

**Affiliations:** 1 Nossal Institute for Global Health and Australia-India Institute, University of Melbourne, Level 5, 333 Exhibition Street, Melbourne, 3000 Australia; 2 Australia India Institute, University of Melbourne, 147-149 Barry Street, Carlton, VIC 3053 Australia; 3 Murdoch Children’s Research Institute, 50 Flemington Rd, Parkville, 3052 Australia; 4 Nossal Institute for Global Health, University of Melbourne, Level 5, 333 Exhibition Street, Melbourne, 3000 Australia; 5 Indian Institute of Public Health-Hyderabad, Plot # 1, A.N.V.Arcade, Amar Co-op Society, Kavuri Hills, Madhapur, Hyderabad, 500 033 India; 6 ‘Pari Mahal’ D-55 Aman Vihar, Lane no.10, Chidowali, P.O:Kandoli, Sahastradhara Road, Dehra dun, Uttarakhand India; 7 CBM, #140, “Commerce Cube”, 5th Main, Puttannachetty Road, Chamarajpet, Bengaluru, Karnataka 560 018 India

**Keywords:** Disability, Inclusion, India, Disabled People’s Organisation, Access, Participation, Wellbeing, Rapid assessment of disability

## Abstract

**Background:**

Disabled People’s Organisations (DPOs) are the mainstay of disability responses worldwide. Yet there is no quantitative data assessing their effectiveness in low-and middle-income countries (LMICs). The aim of this study was to measure the effectiveness of DPOs as a low-cost intervention to improve well-being and access to services and facilities for people with disabilities.

**Methods:**

We undertook a cluster randomised intervention control trial across 39 distinct rural villages in Uttarakhand State, North India. A total of 527 participants were included from 39 villages: 302 people from 20 villages were assigned to the intervention arm and 225 from 19 villages were assigned to the control group. Over a 2-year period, people with disabilities were facilitated to form DPOs with regular home visits. Participants were also given financial support for public events and exposure visits to other DPOs. Seven domains were used to measure access and participation.

**Results:**

DPO formation had improved participation in community consultations (OR 2.57, 95% CI 1.4 to 4.72), social activities (OR 2.46, 95% CI 1.38 to 4.38), DPOs (OR 14.78, 95% CI 1.43 to 152.43), access to toilet facilities (OR 3.89, 95% CI 1.31 to 11.57), rehabilitation (OR 6.83, 95% CI 2.4 to 19.42) and Government social welfare services (OR 4.82, 95% CI 2.35 to 9.91) in intervention when compared to the control. People who were part of a DPO had an improvement in having their opinion heard (OR 1.94, 95% CI 1.16 to 3.24) and being able to make friends (OR 1.63, 95% CI 1 to 2.65) compared to those who were not part of a DPO. All other well-being variables had little evidence despite greater improvement in the DPO intervention group.

**Conclusions:**

This is the first randomised control trial to demonstrate that DPOs in LMICs are effective at improving participation, access and well-being. This study supports the ongoing role of DPOs in activities related to disability inclusion and disability services. This study also suggests that supporting the establishment, facilitation and strengthening of DPOs is a cost-effective intervention and role that non-governmental organisations (NGOs) can play.

**Trial registration:**

ISRCTN36867362, 9th Oct 2019 (retrospectively registered).

## Background

Disability is a complex phenomenon that results from interaction between the person’s impairment and socio-environmental barriers that limit their participation in society [1, 2]. Thus, disability is an umbrella term, covering impairments, activity limitations and participation restrictions [1]. Worldwide, disability prevalence estimates range from 5-25% of the world’s population and approximately 80% of people with disability live in developing countries [1, 3]. In India, the census recorded 2.21% of the population as having a disability or around 26.8 million persons. However, other estimates, using different measures, indicate the figure could be much higher in India [4].

Evidence suggests that people with disabilities are more likely to experience adverse socioeconomic outcomes such as less education, poorer health outcomes, lower education achievements, less employment and higher rates of poverty than persons without disabilities [1, 4, 5]. Additionally, people with disabilities face barriers accessing health and rehabilitation services [2]. In response, disability inclusive development (DID) programs seek to ensure all stages of the development process are inclusive of and accessible to people with disabilities [6]. It goes beyond medical interventions and requires that all persons be afforded equal access to education, health care services, work and employment, and other social activities such as religion and recreation [5, 6]. Low cost and evidence-based interventions are required to promote DID and improve lives of people with disabilities by addressing the socio-environmental barriers.

The United Nations Convention on the Rights of Persons with Disabilities (UNCPRD) expects that people with disabilities should be involved in all elements of the response to disability [7]. ‘Nothing about us without us’ has become the catch phrase of the disability rights movement. Accordingly, in actioning disability inclusive development, people with disabilities should be centrally involved. A central element to involving people with disabilities has been through the formation of Disabled People’s Organizations (DPOs) and Disabled People’s Networks [8]. DPOs grew out of the Disability Rights Movement of the 1970s and have become increasingly mainstreamed in most disability inclusive development programs [9]. DPOs help ensure that development process at all levels are inclusive of the voices and needs of people with disabilities and that they are aware of their rights and participate on an equal basis as others in all aspects of society [6]. DPOs intend to promote participation and well-being through activities such as advocacy, service provision and social support.

The general characteristics of a DPO, although often contested, are that:

- they are established by people with disabilities
- at the board and membership levels, they are controlled by a majority of persons with disabilities (at least 51%) [2, 10];
- they provide persons with disability with “a voice of their own, identifying needs, expressing views on priorities, evaluating services and advocating change and public awareness” [2].

Some studies suggest that organized and registered DPOs working at state or regional level can promote regional cooperation and provide a powerful voice to people with disabilities [11–13]. Whilst increased voice is in itself an important substantive outcome, there is surprisingly little quantitative evidence that DPOs improve well-being and access to facilities and services for people with disabilities in LMICs [8]. A literature review by Young et al found that whilst DPOs could promote well-being, community participation, and rights of people with disabilities [14], there was little published evidence for their impact. While there are some examples of Self-Help Groups (SHGs) tackling some of the barriers facing people with disabilities [15], the disability sector views DPOs as distinct from SHGs and the representation by people with disability is considered of constitutive and instrumental importance [16]. Therefore, it is possible that evidence from a SHG would not be accepted as evidence of impact of a DPO. Furthermore, donors and partners of DPOs would be less likely to accept evidence from SHGs as a rationale for supporting DPOs, or as evidence that DPOs are effective. In fact, there was also a lack of randomised trials examining the effectiveness of DPOs. This is despite the WHO and many aid programs advocating both DPOs and DPO interventions in DID [9, 11].

To be able to measure the impact of DPOs, an effective quantitative tool is required to measure well-being, community participation and access to services. The Nossal Institute for Global Health, with support from Department of Foreign Affairs and Trade (DFAT), Australia and the Centre for Eye Research Australia, has developed the Rapid Assessment of Disability (RAD) survey tool to support the design, implementation and evaluation of DID activities [17]. This sophisticated research tool enables assessment of the impact of an intervention such as DPO formation.

Given the lack of data supporting DPOs, and the availability of an appropriate measurement tool, the primary aim of this study was to measure the effectiveness of DPOs as a low-cost intervention to promote access and well-being. This involved applying the RAD tool before and after the DPO facilitation (intervention), in both the intervention and control groups in Telangana and Uttarakhand. In this paper, we present the study results from the Uttarakhand.

## Methods

A cluster randomized interventional trial was conducted in the Dehradun district of Uttarakhand state in North India to evaluate the impact of DPOs on inclusion, well-being and participation. This study adheres to CONSORT guidelines for reporting clinical trials. The baseline survey was conducted in December 2014 to assess well-being, community participation and access and barriers to services among persons with disabilities [18]. The intervention, partly informed by the baseline results, involved facilitating the formation of DPOs and was carried out between February 2015 and February 2017. The endline survey was conducted over March and April 2017, and involved repeating the same tool used in the baseline on the same participants. Two parallel qualitative research projects were also undertaken to better understand the impacts of DPO and these were published elsewhere [14].

### Sampling technique

A total of 39 villages (or clusters) were purposively selected from 5 distinct areas located in New Tehri and Dehradun districts of Uttarakhand. The project lead in each area selected 5–15 distinct villages to which they had access. Using a lottery system, 20 villages were randomly allocated to the intervention arm and 19 villages to the control arm.

Using an adapted Key Informant (KI) methodology we aimed to identify all people with disabilities from these 39 villages [19]. We consulted a) Government health workers (Anganwadi workers, ASHAs, Village heads), b) School teachers, c) NGO and postal workers, d) Local doctors, e) NGO workers, and f) Religious leaders as the Key Informants who were trained in “what disability is”. All those with disabilities identified were then invited to participate in the study.

### Sample size

All (*n* = 527) people with disabilities in the 39 villages were invited and were surveyed as part of the baseline survey. With 250 samples in each group we could detect a 10% increase in met needs in access to community participation/services or well-being among persons with disability with a statistical power of 74% and confidence interval (CI) of 95%.

### RAD study tool

The RAD survey questionnaire was utilized to evaluate the impact of community led DPOs, by applying the tool before and after intervention, in both the intervention and control groups. The questionnaire was developed by the Nossal Institute for Global Health and the Centre for Eye Research Australia, funded by the Government’s aid programme, to fill a substantial gap in measuring disability. It was developed to identify people at risk of disability in terms of activity limitations and to determine well-being and participation in the community for people with disabilities compared to those without disabilities, and to ascertain associated barriers to participation posed by contextual factors [10]. The questionnaire was developed using two conceptual frameworks: the UNCRPD and the International classification of Functioning, Disability and Health (ICF) [7, 17].

The RAD questionnaire is comprised of elements adapted from existing tools including the Washington Group question set [20] and the Kessler scale. It comprises an interviewer administered household questionnaire and an individual questionnaire. It collects data under five sections: 1) Demographics, 2) Self-Assessment of functioning, 3) Awareness of rights of people with disabilities, 4) Well-being and quality of life, and 5) Participation in the community. Section 2 (self-assessment of functioning) asks about activity limitations over the last six months in seven domains: vision, hearing, communication, mobility, gross and fine motor skills, cognition and appearance. It also includes six questions about psychological distress using an adapted Kessler scale. Response categories were “none”, “some of the time”, and “all of the time”. The RAD tool has been piloted in various settings including in Fiji, the Philippines, and Bangladesh and more recently in India [10, 21–23]. The tool was also used to estimate the prevalence of disability in the two regions, the results of which have already been published [18, 23].

### Data collection

#### Pre- intervention phase: baseline RAD survey (November, 2014)

A baseline RAD survey was conducted amongst people with disabilities from all villages to assess their well-being, community participation and access to services. Informed consent to participate in this study was obtained from all participants. A brief statement in simple, easy to understand English was provided and read out to participants with low literacy. The information about the survey was communicated in ways appropriate for specific disabilities.

#### Intervention phase: formation of disabled People’s Organisations (Feb 2015 – Jan 2017)

In the intervention clusters, local NGOs facilitated the DPO formation by regular home visits to individual families motivating them to be involved in the activities of the DPOs. A curriculum and training program were also developed. Five NGOs, each within one of the intervention sites, were selected to assist in the intervention study and provide support for DPO formation. To ensure all participants in the intervention received the same services or benefits of DPOs, we selected implementing NGOs from the same registered organisation. These NGOs have worked collaboratively for 10 years and have worked closely together on disability for 8 years. We provided the same pre-study training and pilot. Further, all NGOs in the study met regularly during the study.

The participants with disability were encouraged to conduct weekly disability group meetings to facilitate problem solving, advocacy and planning. Every month, half-day training sessions were conducted on the formation of DPO using a DPO manual developed by Community Health Global Network. A regular monthly visit was undertaken by our team to provide ongoing support and encouragement in running twice yearly public events such as the world disability day and a religious festival. As a part of the DPOs, people with disabilities were encouraged to visit the block and district office and make at least three visits to the disability commissioner. Cross exposure visits were also organized, where members of one DPO visited another DPO to organize regular meetings at least monthly and to start new livelihood initiatives in areas of agriculture and horticulture. The interventions were carried out over a two-year duration.

In the non-intervention clusters, no DPOs were facilitated but the ongoing disability work continued. The control group only received referrals for disabilities detected by RAD screening, but without additional assistance. They received the interventions at the end of this study.

#### Post intervention phase- Endline survey (Feb 2017)

The RAD was utilized as an endline survey to assess the impact of DPO intervention on the lives of people with disabilities. Participants in both arms who had been interviewed in the baseline were invited to participate in the endline survey.

### Statistical analysis

The responses for questions related to well-being and access to services were recoded into two groups in line with previously published findings from India [18]. The responses of “All the time” and “Most of the time” were categorised as felt well (well-being section) or Met need (access section). The responses “Some of the time” and “Never” were categorised as Felt unwell (well-being section) or Unmet need (access section). Responses such as “don’t know” or “have not needed” were excluded.

Basic demographic characteristics were summarized using appropriate bi-variate statistics by intervention and control group at baseline and endline separately. All summary statistics were weighted by sampling weights i.e. total sample/number of samples in the cluster. The improvement in outcomes in well-being and access to services due to interventions were examined by Generalised Estimated Equations model. Given the uneven sample sizes of people with disabilities in different villages, we adjusted for the clustering effect in the model. In the first model we estimated the effect of the intervention adjusting for the baseline status while in the second model the implementing NGO was included to adjust for their impact via the intervention on the outcome.

Kobo was used for data collection in the endline and MS Excel was used for cleaning and data management. Stata version 14 was used for data analysis (StataCorp, Texas, USA).

## Results

### RAD survey

**Fig. 1:**
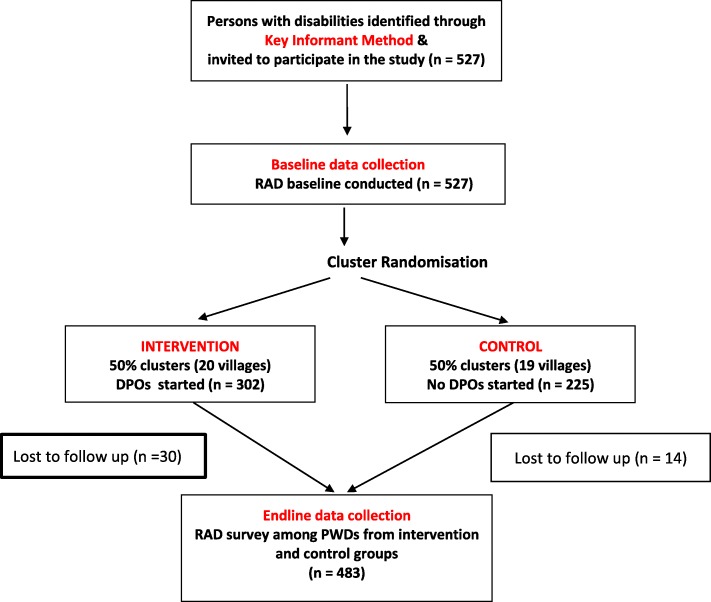
Flow chart of steps in the cluster randomized intervention study

Figure 1 shows the number of participants at each stage of the intervention study. A total of 527 persons with disabilities participated in the baseline RAD survey. Out of these participants 44 (8.3%) were lost to follow-up (Table 1) meaning 483 participants were included in the final analysis.

**Table 1:** Reasons for loss to follow up from baseline to endline

Reasons for loss to follow up	No.
Mismatched B/L to E/L	12
Technical error	3
Died	15
Married & moved away	2
Did not Consent	6
Relocated	2
Could not find	4
Total	44

### Characteristics of study participants

Of the 483 participants, 272 (56%) people with disability were in the intervention group and 211 (44%) in the control group (Table 2). The only significant difference between intervention and control group was in socio economic status (SES) (specially in the middle 40%). The other characteristics were similar between the two groups.

**Table 2:** Demographics of participants in intervention and control groups

	Intervention (*n* = 272)	Control (*n* = 211)
Age (Mean, SD)	40.5 (14.8)	43.4 (15)
Female (n, %)	107 (39.3%)	71 (33.7%)
Married (n, %)	150 (55.2%)	130 (61.6%)
Ever attended school (n, %)	166 (61%)	124 (58.8%)
Socio Economic Status^ab^		
Lowest 40%	100 (37.6%)	89 (43.4%)
Middle 40%	120 (45.1%)	68 (33.2%)
Highest 20%	46 (17.3%)	48 23.4%)

^a^Socio-economic status was based on total composite SES scores derived from household variables (land ownership, type of brick walls, floors, fuel, assets, electricity, stock and microcredit)

^b^Intervention group: *n* = 266; control group: *n* = 205

### Well-being of people with disabilities

Participants in the intervention group demonstrated an increase in the positive responses (met needs) between the baseline and endline survey across all well-being variables (Table 3).

**Table 3:** Changes in Well-being indicators in the control and intervention groups

Well-being	% of met needs at baseline^a^ (n; 95% CI)		% of met need at endline^a^ (n; 95% CI)	
	Intervention	Control	Intervention	Control
Confident	46% (99, 35.7 to 56.7%)	48.5% (72, 33.5 to 63.8%)	59.9% (130, 46.6 to 71.8%)	45.1% (77, 21.8 to 70.7%)
Respected by community	81.4% (191, 66.2 to 90.7%)	75.3% (124, 64.6 to 83.5%)	84.3% (211, 73.5 to 91.2%)	82.8% (153, 69.1 to 91.1%)
Opinion	67% (154, 60 to 73.4%)	63.1% (104, 55.9 to 69.7%)	75.6% (179, 66.4 to 82.9%)	62.1% (117, 52.5 to 70.9%)
Able to make friends	23.9% (55, 11.4 to 43.5%)	18.2% (37, 8 to 36.3%)	37.8% (89, 20.3 to 59.1%)	32.4% (55, 15.7 to 55.4%)
Living condition	75.6% (181, 54.2 to 89%)	69% (130, 49.3 to 83.6%)	91.1% (237, 81.4 to 96%)	82.3% (163, 68.2 to 91%)
Help Others	20.1% (41, 7.4 to 44.1%)	11.1% (25, 5.5 to 21.4%)	34.3% (76, 16.4 to 58.2%)	32.4% (52, 10.9 to 65.2%)

^a^Percentages were weighted by sampling weights i.e. total sample / number of sample in the cluster

Most of the well-being items in model 1 demonstrated relatively higher odds of met needs in the intervention group than in the control group (Table 4). The cluster-adjusted odds ratios were all above 1 after controlling for baseline status. In the second model, in which implementing NGO was added as a covariate, ‘opinion being considered’ (OR 1.94, 95% CI 1.16 to 3.24, *p* = 0.01) and ‘being able to make new friends’ (OR 1.63, 95% CI 1 to 2.65, *p* = 0.05) displayed positive association between intervention and met needs. Both suggested the odds of met needs (able to make new friends and opinion were considered) in the intervention group were approximately twice that of the control group.

**Table 4:** Association between intervention and well-being

WELL-BEING	n	Model 1^a^		Model 2^b^	
		Intervention Odds Ratio (95% CI)	*p*-value	Intervention Odds Ratio (95% CI)	*p*-value
Confident	370	1.12 (0.5 to 2.6)	0.78	1.64 (0.9 to 2.98)	0.15
Respected by community	402	1.36 (0.58 to 3.22)	0.48	1.34 (0.6 to 3.0)	0.47
Opinion	399	1.62 (0.85 to 3.1)	0.14	1.94 (1.16 to 3.24)	0.01
Able to make friends	341	1.3 (0.45 to 3.79)	0.63	1.63 (1 to 2.65)	0.05
Living condition	433	2.28 (0.96 to 5.41)	0.06	2.01 (0.89 to 4.62)	0.09
Help Others	380	0.83 (0.24 to 2.86)	0.76	1.28 (0.7 to 2.33)	0.42

^a^Explanatory factors: group and baseline; adjusted for clusters (ADPs)

^b^Explanatory factors: group, baseline and NGO; adjusted for clusters (ADPs) Chamba (NGO) was the reference group in the GEE model 2 with implementing NGO added as a covariate

### Access to services

In regard to the access and participation variables there were consistently more individuals in the intervention groups who reported their needs were met in the endline compared with baseline (Table 5). Again, the increases in percentages of met needs were more substantial across all participation variables in the intervention group, except access to toilet facilities of which the increases were approximately the same in the two groups. In almost all the access to services/facilities items in the control group, the 95% confidence intervals of proportions of the two time points overlapped. This indicates that there was not enough evidence of a difference in proportions between baseline and endline.

**Table 5:** Changes in Access to services and facilities, and Community Participation in control and intervention groups

Access and Participation	% of met needs at baseline^a^ (n, 95% CI)		% of met need at endline^a^ (n, 95% CI)	
	Intervention	Control	Intervention	Control
Work	63.3% (119, 52.5 to 73%)	75.4% (116, 55.1 to 88.4%)	81.5% (166, 72.2 to 88.2%)	74.9% (128, 54.4 to 88.2%)
Community Consultation	58.9% (99, 39.4 to 75.9%)	65% (78, 38.1 to 84.9%)	67.3% (124, 51.4 to 80%)	58.8% (76, 26.7 to 84.8%)
Rehabilitation services	19% (27, 12.3 to 28.3%)	43.2%(40, 33.8 to 53.1%)	70% (45, 47.8 to 85.6%)	30% (17, 20 to 42.4%)
Access to safe drinking water	86.6% (210, 77.4 to 92.4%)	88.7% (159, 72.6 to 95.9%)	90.8% (240, 85.1 to 94.5%)	87.5% (179, 80 to 92.5%)
Access to toilet facilities	92.4% (234, 87.5 to 95.4%)	95.2% (183, 82.3 to 98.8%)	96.9% (263, 94.3 to 98.4%)	93.6% (201, 90.2 to 95.9%)
Able to participate in social activities	70.8% (147, 55.3 to 82.5%)	85.3% (117, 69.2 to 93.8%)	81.6% (185, 66.2 to 91%)	77.7% (125, 54.7 to 90.9%)
Access to Govt. social welfare services	69.8% (129, 60.8 to 77.5%)	80.1% (106, 69.2 to 87.8%)	89.4% (201, 78.6 to 95.1%)	79.1% (126, 57.2 to 91.4%)
Access to DPO	15.4% (14, 6 to 34.2%)	24.4% (12, 19.7 to 29.8%)	56.7% (125, 40.1 to 71.9%)	22.4% (11, 5.2 to 60.3%)
Access to legal Aid	70.5% (21, 44 to 87.9%)	90.3% (34, 61.5 to 98.2%)	87.3% (34, 56.3 to 97.3%)	77.5% (24, 26.9 to 97%)

^a^Percentages were weighted by sampling weights i.e. total sample / number of sample in the cluster

When we applied the Generalised Estimated Equations, accounting for the cluster effect and the effect of the implementing agencies, nearly all the variables showed evidence of improvement in participation (Table 6). That is, after controlling for implementing agencies, the odds of met needs for these variables in the intervention group were at least 2.6 of those not in the intervention program.

**Table 6:** Association between intervention and access/participation outcomes

Access and participation	n	Model 1^a^		Model 2^b^	
		Intervention Odds Ratio (95% CI)	*p*-value	Intervention Odds Ratio (95% CI)	*p*-value
Work	295	0.66 (0.26 to 1.69)	0.39	0.76 (0.32 to 1.79)	0.53
Community Consultation	255	1.83 (0.68 to 4.93)	0.23	2.57 (1.40 to 4.72)	0.002
Rehabilitation services	71	6.69 (2.42 to 18.48)	0.00	6.83 (2.4 to 19.42)	< 0.001
Access to safe drinking water	444	1.45 (0.71 to 2.94)	0.30	1.53 (0.84 to 2.78)	0.16
Access to toilet facilities	400	2.75 (0.81 to 9.37)	0.02	3.89 (1.31 to 11.57)	0.01
Able to participate in social activities	327	2.46 (0.99 to 6.11)	0.05	2.46 (1.38 to 4.38)	0.002
Access to Govt. social welfare services	295	3.53 (1.29 to 9.69)	0.01	4.82 (2.35 to 9.91)	< 0.001
Access to DPO	69	12.24 (1.54 to 97.29)	0.02	14.78 (1.43 to 152.43)	0.01
Access to legal Aid	17	1.13 (0.86 to 1.5)	0.38	NA	NA

^a^Explanatory factors: group and baseline; adjusted for clusters (ADPs)

^b^Explanatory factors: group, baseline and NGO; adjusted for clusters (ADPs) Chamba (NGO) was the reference group in the GEE model 2 with implementing NGO added as a covariate

Out of the 6 access variables, only access to work and access to safe drinking water showed little evidence of a difference between intervention and control groups. Access to legal aid could not be tested using the GEE due to extremely small numbers.

## Discussion

This is the first randomised control trial in any LMIC to quantitatively demonstrate the impact of DPOs – a key element of the disability response. The study shows how an intervention to facilitate DPOs may lead to significant improvement in many areas of well-being and access to services and facilities. DPOs were demonstrated to be associated with substantial improvement in access to most of the services and facilities, whereas sense of well-being was improved in two of the six variables. This study has significant implications for the practice of disability inclusive development in terms of advocating for the role of DPOs and generating further support for them. These results likely underestimate the DPO’s effect for those individuals who are participating in the DPO because this study measured the DPO’s impact on all people with disabilities in the community; not just those who participated in the DPO, although the odds ratio of 15 for access to DPO seems to indicate that many of the study participants were accessing the DPO.

Research has shown that people with disabilities are more likely to have smaller and less diverse social networks compared to people without disabilities [24–26]. Almost by definition the social network for people with disabilities was expanded through the intervention which involved the formation of a DPO. Indeed the parallel qualitative studies [14] (published elsewhere) indicated that participation in the DPO, as expected, has increased the social networks of people with disabilities. Social Network Analysis is currently being undertaken to map out the social networks of the DPO participants, before and after the DPO formation. Growing the social network is important as the literature indicates that social networks, in themselves, are important indicators for an individual’s health and well-being [27, 28]. Intuitively increased access to rehabilitation services, toilet facilities and social welfare program might expect to lead to improved health outcomes for those with disabilities. Although change in health status was not measured, the multidimensional well-being and improvements were observed in some domains.

The overall budget of the DPO intervention was around USD $18,000 per year across 25 villages. This small investment has resulted in a widespread gain across access, participation and well-being for people with disabilities. Therefore, this intervention, and the formation and support of DPOs, seemingly represents a highly cost-effective investment.

However, improvement was not seen in all domains. Some national programs and insurance programs in disability, such as the National Disability Insurance Scheme (NDIS) in Australia, are predicated on the relationship between increased access to services/care resulting in increased employment of people with disabilities and, in turn, increased Gross Domestic Product (GDP). However, in this study, despite increased access to services and rehabilitation, employment was the access variable that did not show any improvement. Nonetheless, access to work is a more distal outcome, which over time, might be expected to increase due to increased well-being and access to services. Additionally, the way that the question was asked “do you have as much access to work as you would like” may have not captured increase work in the informal sector. For example, even if the intervention increased engagement in domestic and subsistence work (not salaried), this would probably not have been reported as “access to work”.

The loss to follow up was acceptable (8%) and the most common factor for failure to follow up was the death of the participants (*n* = 15). In a study population of 527 people this death rate (14.2 deaths per 1000 population per year) is dramatically higher than expected for adult males in India for whom the crude mortality rate is 7.3 deaths per 1000 population/year [29] Higher death rates among people with disabilities are observed worldwide, and more markedly in LMICs. Decreasing mortality was not an outcome measure in this study but, given enough time, one would anticipate that the increase in access and well-being might translate to decreasing this high mortality rate.

The context of the DPO formation seems to be important as indicated by the implementing partner, or NGO, being a confounder which when controlled for changed the findings. Therefore, the way that a DPO is facilitated and supported seems to be important for its success and this finding was supported from the parallel qualitative study undertaken by Young et al. [14]. This raises the contention as to how much external support and facilitation is helpful or necessary for DPOs. From a disability rights perspective, DPO should be conceived of, planned, initiated, led and operated exclusively by people with disabilities. Yet in this study the level and quality of external supports seems to be important for the establishment and effectiveness of the DPO.

Acknowledging that the context is important, the qualitative study [30] and realist review [14] conducted in parallel to this quantitative study are important for understanding the specific context for the changes. This provides useful learnings as to how and why the DPO worked in this setting. This contextual information from the qualitative work may help the reader determine if and/or how to undertake this intervention in other contexts.

### Limitations

The generalizability of these findings is limited given this study was in only two districts. The study was perhaps statistically underpowered to detect changes in the well-being domains. All well-being indicators improved in the intervention group more than in the control group arm yet only two of six well-being variables showed enough evidence of a difference. This seemingly relates to the sample size indicated by a wide 95% confidence interval of odds ratio for ‘living conditions’ (0.89 to 4.62, *p* = 0.09) and ‘confidence’ (0.9 to 2.98, *p* = 0.15).

The ratio of males (*n* = 305) to females (*n* = 178) is seemingly skewed. However, this is partly explained by a higher prevalence of disability in males (8.1%) than in females (6.7%) in these districts in Uttarakhand [18]. If we apply these prevalence estimates to our study sample, then we would expect 264 males and 219 females. While this may indicate an under-representation of women with disabilities in our sample, this also may reflect the reality of entrenched inequalities facing women with disabilities: in India, women are often given less value in a society. It is possible that women with disabilities were less visible or excluded, making it more challenging for our informants to identify women with disabilities [19]. To address this in the future research, it may be beneficial to enlist support from local women’s network to effectively encourage women with disabilities to part-take in surveys and to ensure studies are carried out in the most considerate, sensitive way.

## Conclusion

This study is the first to provide quantitative evidence that DPOs are in fact effective at increasing participation, access and well-being. DPOs are the mainstay of disability responses worldwide and a key element for the disability rights movement [7]. This study supports the ongoing role of DPOs and suggests that investing in supporting their establishment, facilitation and strengthening is a cost-effective intervention. Government programs should continue to work closely with the NGOs and community-based organisations to strengthen DPOs. This could include undertaking training, building technical capacity building in running a society, lining them with appropriate NGOs. A future study is planned to assess the sustainability of the effects demonstrated.

## Data Availability

The datasets used and/or analysed during the current study are available from the corresponding author on reasonable request.

## Abbreviations

DFAT: Department of Foreign Affairs and Trade
DID: Disability inclusive development
DPO: Disabled People’s Organisation
GDP: Gross Domestic Product
ICF: International classification of Functioning, Disability and Health
KI: Key Informant
LMIC: Low-and Middle-Income Country
NDIS: National Disability Insurance Scheme
NGO: Non-Governmental Organisation
RAD: Rapid Assessment of Disability
SES: Socio-Economic Status
SHG: Self-Help Group
UNCRPD: The United Nations Convention on the Rights of Persons with Disabilities
